# The National Cancer Institute’s Co-Clinical Quantitative Imaging Research Resources for Precision Medicine in Preclinical and Clinical Settings

**DOI:** 10.3390/tomography9030076

**Published:** 2023-04-30

**Authors:** Huiming Zhang

**Affiliations:** Cancer Imaging Program, Division of Cancer Treatment and Diagnosis, National Cancer Institute, NIH, Bethesda, MD 20892, USA; zhanghui@mail.nih.gov

**Keywords:** co-clinical imaging, co-clinical trial, quantitative imaging, co-clinical imaging research resource, PDXs, GEMMs, CIRP web resource, preclinical quantitative imaging

## Abstract

Genetically engineered mouse models (GEMMs) and patient-derived xenograft mouse models (PDXs) can recapitulate important biological features of cancer. They are often part of precision medicine studies in a co-clinical setting, in which therapeutic investigations are conducted in patients and in parallel (or sequentially) in cohorts of GEMMs or PDXs. Employing radiology-based quantitative imaging in these studies allows in vivo assessment of disease response in real time, providing an important opportunity to bridge precision medicine from the bench to the bedside. The Co-Clinical Imaging Research Resource Program (CIRP) of the National Cancer Institute focuses on the optimization of quantitative imaging methods to improve co-clinical trials. The CIRP supports 10 different co-clinical trial projects, spanning diverse tumor types, therapeutic interventions, and imaging modalities. Each CIRP project is tasked to deliver a unique web resource to support the cancer community with the necessary methods and tools to conduct co-clinical quantitative imaging studies. This review provides an update of the CIRP web resources, network consensus, technology advances, and a perspective on the future of the CIRP. The presentations in this special issue of Tomography were contributed by the CIRP working groups, teams, and associate members.

## 1. Introduction

The National Cancer Institute (NCI; Bethesda, MD, USA) moved into the forefront of precision medicine in 2014 [1]. A substantial increase in support was provided to the cancer community to develop new laboratory models, enhance treatment resistance research, expand precision medicine clinical trials, and establish a national cancer knowledge system [1]. Genetically engineered mouse models (GEMMs) and patient-derived xenograft mouse models (PDXs) are now being extensively utilized in therapeutic trials to recapitulate the biology and phenotypes of human cancers. They are often part of precision medicine studies in a co-clinical setting in which therapeutic investigations are conducted in patients and in parallel (or sequentially) in cohorts of GEMMs or PDXs. These preclinical investigations can supplement prospective or retrospective human clinical trials. Employing radiology-based quantitative imaging in these studies allows in vivo assessment of disease response in real time, providing an important opportunity to bridge precision medicine from the bench to the bedside. Historically, in the pipeline of imaging methodology development, preclinical imaging was relegated to exploratory, proof-of-concept studies. Preclinical imaging methods remained as “home-built”, laboratory-centric approaches, qualitative or semi-quantitative, without universal standards or protocols. NCI initiated the Co-Clinical Imaging Research Resource Program (CIRP) in 2015 to establish web-accessible, co-clinical, quantitative imaging research resources funded under several program announcements [2]. Each CIRP resource is required to incorporate four essential components, including animal models (GEMMs or PDXs), co-clinical therapeutic trials, quantitative preclinical and clinical imaging methods, and informatics for supporting web resources. The intent of this review is to provide an update of NCI’s CIRP web resources, network consensus, technology advances, and provide a future perspective.

## 2. Historical Background

GEMMs or PDXs can recapitulate important biological features of cancer and played important roles in advancing precision medicine. To this end, NCI supported the development of mouse models via the Mouse Models of Human Cancers Consortium program (MMHCC, 1998–2014) [3]. One important result of the MMHCC projects was the formulation of the co-clinical trial concept [4]. Continuous support by NCI includes the Oncology Model Forum (OMF) program in 2014, made to establish a research resource for a variety of animal models [5], the Patient-Derived Model of Cancer (PDMC) program in 2016, made to develop xenograft models [6], the PDX development and Trial Centers Research NETwork (PDXnet) program in 2017, made as a resource for therapeutic development [7], and the Patient-Derived Models Repository (PDMR), made in 2017 to create a repository for patient-derived models (PDMs) comprised of patient-derived xenografts (PDXs), tumor cell cultures, cancer associated fibroblasts, and patient-derived organoids [8]. NCI also supported the establishment of PDX and/or GEMM models of pediatric cancers for therapeutic development research. These include the Pediatric Preclinical Testing Consortium (PPTC) in 2014–2019 and the Pediatric in Vivo Testing Program (PIVOT), established in 2020 [9]. PDX and GEMM models often involve cancer in organs or at orthotopic sites deep in the mouse body, requiring robust imaging approaches to determine tumor growth, burden, and therapeutic response quantitatively. Many therapeutic studies utilize multiple animal model cohorts to capture the biological heterogeneity of human diseases and to obtain a therapeutic response time-course. Animal studies require that imaging acquisition must have good repeatability and reproducibility during repeat imaging sessions at different timepoints.

Radiology imaging methods can provide a non-invasive, quantitative assessment of cancer stage and treatment response via measuring alterations in anatomy, physiology, perfusion, cellularity, vascularity, metabolism, and specific biomarkers of interest. To ensure the quality of quantitative imaging biomarkers for clinical trials, stakeholders invested efforts to establish methods and tools for quality assurance/control (QA/QC) and robust data analysis, to develop harmonization and standardization across sites for broad adoption and implementation of SOPs in clinical trials, and to establish imaging archive resources [10]. NCI supported several initiatives to address these critical needs in clinical quantitative imaging. These include the Quantitative Imaging Network (QIN) for optimizing imaging protocols and tools since 2008 [11], the Cancer Imaging Archive (TCIA) for providing public access to clinical imaging data sets since 2011 [12], and the Imaging Data Common (IDC) for image repository since 2019 [13]. Meanwhile, NCI’s Informatics Technology for Cancer Research (ITCR) program supported the development of imaging-specific informatics tools and resources since 2012 [14]. Due to the limited number of preclinical trials prior to the availability of large PDXs cohorts, there is little support for the standardization or harmonization of preclinical imaging methods to provide reliable, quantitative biomarkers for preclinical trial studies.

Preclinical quantitative imaging of cancer animal models faces different challenges compared to the clinical quantitative imaging of cancer patients. GEMM and PDX mouse models (body weight in grams) are very small compared to the human (body weight in kg). Imaging of in situ tumors in GEMMS or orthotopic tumors in PDXs is technically challenging in the context of imaging quality and detection sensitivity. Both are dependent on the size of the tumor and the location of the tumor in the body. Several issues are critical for obtaining reliable preclinical quantitative images, including imaging technology, motion artifacts, and data processing. To conduct multi-cohort and/or multi-site preclinical imaging studies successfully, the imaging methods need to be harmonized and standardized in the context of imaging protocols and software tools, metadata format, methodology interoperability, data sharing, and achieving. With the use of multi-cohort mouse models in precision medicine research at multi-sites, the development of preclinical quantitative imaging methods and establishment of related SOPs became an emerging need for preclinical trials. For this reason, NCI’s CIRP program was launched in 2015 to fill this gap through the establishment of research resources for both preclinical and clinical settings, providing these co-clinical imaging research resources to the cancer community [15]. This program had active FOAs from 2015 to 2021 and funded a total of 10 different projects spanning diverse tumor types, therapeutic interventions, and imaging modalities.

## 3. NCI’s CIRP Program

**Figure 1:**
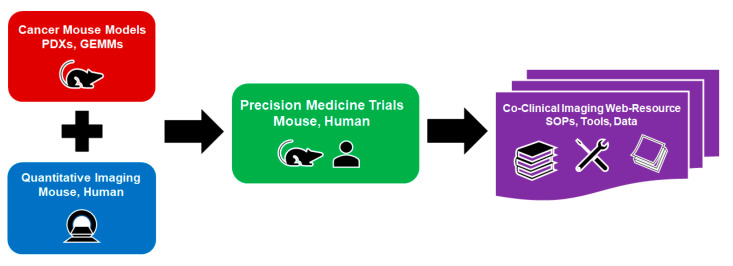
Four essential components of the co-clinical imaging research resource are presented in color-coded boxes: cancer mouse models that include available, credentialed PDXs, and/or GEMMs (red), quantitative imaging methods that are well-matched for mouse and human application (blue), co-clinical trials with matched therapeutic intervention for mouse and human (green), and a web resource to publicly share all standard operating procedures (SOPs), tools, and data (purple).

The scientific objectives of the CIRP are to provide the cancer community with web-accessible research resources for quantitative imaging of co-clinical trials, and to encourage consensus on how quantitative imaging methods can be optimized to improve the quality of imaging results for co-clinical trials. As shown in Figure 1, a CIRP resource comprises four essential components: (1) cancer mouse models, (2) quantitative imaging, (3) co-clinical trials, and (4) informatics for establishing a web resource. The animal models make use of credentialed GEMMs or PDXs to re-capitulate the biological features of cancer to address important issues for adult or pediatric cancer. The co-clinical trials take advantage of known therapeutic interventions to optimize quantitative imaging methods. The preclinical quantitative imaging methods are either through reverse translation of clinical quantitative imaging methods to preclinical imaging methods, or prospectively pairing FDA-approved, new clinical imaging methods with matched preclinical imaging methods. The emphasis of both approaches focuses on the optimization of the preclinical imaging methods. The informatics component employs state-of-the-art methods for archiving images and metadata, tool sharing, and providing user-friendly CIRP web accessibility. Each CIRP project is required to target a specific cancer issue for which the co-clinical quantitative imaging may play a critical role. Though hypotheses may be included, the funded projects are primarily focused on research resource development and dissemination.

**Figure 2:**
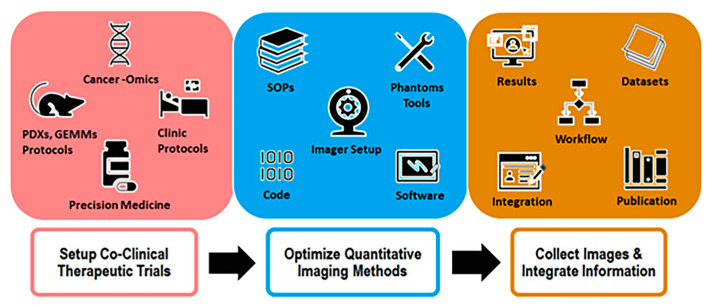
Diagram of the CIRP project workflow.

The workflow of a typical CIRP project is schematically shown in Figure 2. The first step is to establish a co-clinical trial with appropriate PDXs or GEMMs credentialed with cancer-omics, and then design the preclinical intervention matched to the clinical intervention. The second step is to optimize preclinical quantitative methods to establish imaging SOPs, QA/QC tools, and the appropriate data processing software. The third step is to apply the optimized preclinical imaging methods and the matched clinical imaging methods to collect co-clinical images, thereby enabling multi-scale data integration. Meanwhile, a web portal is established to populate the obtained functional information for dissemination, including all methods, SOPs, tools, and representative data for biology, pathology, and preclinical and clinical images. With this workflow, the project needs to be organized around approaches that integrate the four essential components together.

The deliverable of a CIRP-funded project is a web resource populated with all the methodologies, protocols, tools, and data associated with the quantitative imaging in preclinical and clinical trial settings by the end of year 5. Explicitly, these include the protocols for animals, biology/pathology/histology, imaging and QA/QC, software for imaging reconstructions, data processing and analysis, representative data for biology, pathology, and preclinical and clinical images. Though each project may generate multi-scale data ranging from cancer-omics to tumor pathology and from in vivo preclinical images to clinical images, the integration of biologic information with imaging information is not a primary required deliverable. The CIRP as a network program has intra-network working groups (WGs) to develop a consensus on the best means for designing and the implementation of quantitative imaging methods for the planned co-clinical trials, the development of web-accessible research resources, and the strategy of outreach to the research community. The WGs’ effort is also focused on ensuring that the best practices are applied to the functional information collection and dissemination for the CIRP web resources.

## 4. The CIRP Projects

The CIRP program awarded ten 5-year projects between 2017 and 2022. As listed in Table 1, these projects include seven adult and one pediatric cancer, including breast, bone, colon, hematology, lung, muscle, pancreas, and prostate cancers. Seven projects are focused on primary tumors, and three on metastatic tumors. Regarding the animal models utilized, six projects use PDXs, and four use GEMMs. All the co-clinical trials are therapeutic trials, with nine prospective trials and one retrospective trial. Among the prospective trials, four are initiated by the investigator’s institutions, two are supported by NCI’s Specialized Program of Research Excellences (SPOREs) [16], one is sponsored by NCI’s Cancer Therapy Evaluation Program (CTEP) [17], and two are sponsored by consortia within the cancer community. The co-clinical trials utilize chemotherapy (three), targeted therapies (two), combined targeted/immunotherapy (two), combined radiation/immunotherapy (one), immunotherapy (one), and hormone therapy (one).

The quantitative imaging methods employed in these projects include MRI, MRSI, PET, CT, MRI/PET, and PET/CT to assess alterations in anatomy, metabolism, cellularity, vascularity, perfusion, and specific metabolic and molecular targets. Specific metabolic and molecular targets that are being imaged using PET include glucose uptake and glycolysis using ^18^F-fluorodeoxyglucose (^18^F-FDG), glutamine uptake and glutaminolysis using (4*S*)-4-(3-^18^F-fluoropropyl)-L-glutamic acid (^18^F-FSPG) and ^18^F-4-fluoro-glutamine (^18^F-Gln), and estrogen receptor (ER) status using ^18^F-fluoro-furanyl-norprogesterone (^18^F-FFNP). Specific molecular and metabolic targets that are being imaged using MRI are tumor-associated macrophages using ferumoxytol and metabolic fluxes in glycolysis (pyruvate conversion to lactate) using hyperpolarized (HP) ^13^C-pyruvate. The CIRP projects are also improving the quantitative imaging sensitivity and quality of mouse tumors by using more sensitive hardware and imaging techniques. Examples of these include the use of a cryo-cooled MRI radiofrequency coil to increase the signal to noise ratio (SNR) of bone marrow images of mouse tibia, and the use of the radial *k*-space sampling image method to reduce motion artifacts in mouse pancreas images. Additionally, two CIRP projects are exploring strategies for information integration from biology to imaging across species spatially and temporally.

All CIRP projects are targeted at specific cancer issues, using co-clinical imaging as a tool to bridge gaps between preclinical trials and clinical trials via different pathways. Examples are a co-clinical approach to conduct preclinical and clinical trials in parallel, a sequential approach to supplement clinical trials with preclinical trials, or a retrospective approach to mimic clinical trials with preclinical trials. For myelofibrosis, a combination of quantitative MRI biomarkers, including fat fraction (FF), apparent diffusion coefficient (ADC), and magnetization transfer ratio (MTR), is used to assess bone marrow status, which may replace invasive sequential bone marrow (BM) biopsies. In this project, the preclinical imaging study of GEMMs is feedbacked to the clinical investigations to accelerate therapeutic development. For pancreatic ductal adenocarcinoma (PDAC), MRI biomarkers *K^trans^* and *ADC* are used to characterize the status of pancreatic stroma in the co-clinical setting for assessing the therapeutic efficacy of combined stroma-directed therapy and chemotherapy. For non-small cell lung cancer (NSCLC), co-clinical ^18^F-FDG PET imaging is employed to assess the therapeutic response of combined anti-programmed death ligand 1 (PD-L1) immunotherapy and C-X-C motif chemokine receptor 2 (CXCR2) target therapy. In this project, the glucose uptake is used as a biomarker to evaluate the response of the combined therapy that blocks recruitment of monocytes and neutrophils into a tumor microenvironment. For small cell neuroendocrine prostate cancer (SCNC), SCNC PDXs with tumor metastasis in the liver and bone are used in the matched preclinical trial. HP 13C MRI is employed to measure the rate of conversion HP 13C pyruvate to HP lactate (*k*_pl_) in real time to provide an early quantitative assessment of the therapeutic response at liver and bone metastatic sites, simultaneously.

## 5. The CIRP Web Accessible Resources

The CIRP program has a central webpage at https://imaging.cancer.gov/, accessed on 20 April 2023 [15] that provides a collection of hyperlinks to the CIRP project abstracts and the CIRP web resources. All CIRP projects launched a website for their web resources (Table 1), which are expected to be completed during the 2022 to 2027 timeframe. The triple-negative breast cancer (TNBC) project of Washington University in St Louis (WUSTL) and the soft tissue sarcoma project at Duke University (Duke) were completed in 2022.

The focus of the WUSTL project is to develop bi-directional pipelines for quantitative imaging, radiomics, and genomics in a co-clinical setting. These pipelines will be used to assess tumor heterogeneity and predict therapeutic response in breast cancer. The WUSTL web resource (https://c2ir2.wustl.edu/, accessed on 20 April 2023) is divided into four sections: Research, Publications, Resources, and a Co-Clinical DataBase (CCDB). The Research section provides an overview of the TNBC project, the relevant radiomics, and genoproteomic discovery. The Resources include QA/QC phantoms for preclinical ADC MRI and dynamic contrast enhanced (DCE) MRI, PDXs models, SOPS for TNBC PDXs, SOPs for daily/weekly and monthly scanner QA/QC, software/code for automatic segmentation of PDX tumors, a PET hotel session splitter for multiple-mouse imaging, and representative datasets. Representative publications include a test–retest result of TNBC PDXs using a multiparametric MR acquisition protocol [18], reproducibility of preclinical PET response criteria in solid tumors (PERCIST) for TNBC tumors [19], optimal co-clinical radiomics for MR images [20], deep learning segmentation for TNBC PDX MRI data [21], and co-clinical FDG-PET radiomic signatures for predicting TNBC response to neoadjuvant chemotherapy [22].

The purpose of the Duke project is to optimize preclinical MRI protocols of the extremity and respiratory-gated CT protocols of the lungs to assess the therapeutic response of soft tissue sarcoma GEMMs being treated with combined radiation therapy and PD-1 inhibitor immunotherapy. The Duke web resource (https://sites.duke.edu/pcqiba/, accessed on 20 April 2023) comprises five sections, including MRI, Micro-CT, Protocols, Code, and Data and Publications. The Protocols section includes SOPs for micro-CT of lung nodules, SOPs for MRI at 7T, protocols for tumor segmentation using a 3D Slicer, and protocols for primary sarcoma GEMM generation. The Code section includes a MATLAB-based tool for converting 3D image volumes from the Neuroimaging Informatics Technology Initiative (NifTi) format to the Digital Imaging and Communications in Medicine (DICOM) format, as well as code for tumor segmentation, radiomics analyses, and deep learning lung nodule detection. Representative publications include respiratory-gated micro-CT imaging for the detection of murine lung tumors [23], micro-CT and micro-MRI protocols for tumor burden assessment in a co-clinical trial [24], MRI-based deep learning segmentation and radiomics of murine sarcoma [25], connection of murine sarcoma MRI and histology [26], and deep learning micro-CT lung nodule detection [27].

## 6. The CIRP Network Consensus

The CIRP network has three WGs, including the animal models and co-clinical trials (AMCT) WG, imaging acquisition and data process (IADP) WG, and InforMatics and OutReach (IMOR) WG. The AMCT WG seeks to develop a consensus in best practices for employing animal models in co-clinical trials. The IADP WG is focused on developing a consensus for designing, optimizing, and applying best practices for preclinical quantitative imaging to support co-clinical trials. The IMOR WG facilitates the creation of a web-accessible research resource to include data, methods, workflow documentation, and results collected from the co-clinical investigations. The CIRP network also includes six NIH-supported investigators/scientists as associate members. These associate members brought their expertise and effort to the WGs to expand the scientific scope of the network and achieve broad consensus on important issues in co-clinical imaging.

The CIRP network worked to reach consensus on key issues since 2019, including animal model selection, co-clinical study design, standardization of co-clinical instruments, harmonization of co-clinical imaging acquisition, and data analysis pipelines [28]. This special issue provides a snapshot of current issues and consensus that were explored by the WGs [29,30,31,32]. The AMCT WG conducted a survey across the CIRP network to identify challenges in using animal models, including PDXs, GEMMs, and transplantable mouse models in co-clinical trial settings, to seek consensus on best practices for employing animal models in co-clinical imaging [29]. The IADP WG developed preclinical imaging protocols (PIPs) templates for CT, PET, and MRI. An online repository for PIPs was established at the protocols.io [30] to standardize imaging protocols and improve reproducibility in preclinical imaging. The PIPs include preclinical imaging claims, imaging process specification, animal model specification, quantitative metrics, and supporting data. The IADP WG also completed a multi-site ADC phantom study on 10 preclinical MRI systems (seven sites, three vendors, and six field strengths), to establish the accuracy (bias), precision (repeatability), and reproducibility of preclinical ADC MRIs [31]. The IMOR WG collected descriptions of the CIRP projects and defined a list of data collection items for CIRP web resources, which are publicly available at the CIRPHub (https://ncihub.org/groups/cirphub/collections/cirp-web-accessible-resources, accessed on 20 April 2023). The IMOR WG also conducted a survey on small animal DICOM and metadata to identify co-clinical imaging minimal information (CIMI) required for the preclinical imaging. The CIMI provides a critical list of information for enhancing the reproducibility of preclinical imaging and enabling search and query of a database to support data mining and analytic pipelines [32].

## 7. Advanced and Emerging Methods

To improve the quality of imaging results for co-clinical trials, the CIRP projects are required to overcome technical challenges for performing quantitative imaging in the preclinical setting. These challenges include increasing SNR, reducing motion artifacts, optimizing protocols for improving repeatability and reproducibility, and improving sensitivity and specificity. Several manuscripts in this special issue contributed by the CIRP teams present the progress on the development of new technology and methodology [33,34,35,36,37,38,39,40].

MR imaging the BM in the mouse tibias is challenging due to the small size (1 mm diameter) that limits the SNR, and thus the accuracy of quantitative imaging biomarkers. Using a cryo-cooled MRI to receive coil for SNR improvement, Ross et al. successfully investigated the repeatability measures of quantitative imaging metrics for the tibia GEMMs of myelofibrosis, including FF, ADC, and MTR methods [33]. MR imaging of PDAC in GEMMs suffers from respiratory motion and increased magnetic susceptibility artifacts at high magnetic fields. Using the radial *k*-space sampling technique, Pickup et al. demonstrated that respiration artifact-free images with good SNR are obtained for orthotopic PADC in GEMMs, leading to robust estimation of quantitative metrics from DCE MRI [34]. Dynamic hyperpolarized ^13^C metabolic imaging can provide a quantitative assessment of the pyruvate to lactate conversion rate, *k*_pl_, as a biomarker for cancer aggressiveness and therapeutic response. To improve SNR and rate constant fitting precision, Sahin et al. reversely engineered a 2D metabolite-specific clinical EPI protocol to a preclinical imaging system and tested it on in vivo mice with renal cell carcinoma (RCC) PDXs and prostate cancer PDXs [35]. For evaluation of combination therapy, Bae et al. tested ^18^F-FSPG for predicting early response to combined inhibition of EGFR and glutaminolysis in RAS colorectal cancer (CRC) PDXs [36]. Co-clinical PET imaging of immune checkpoint inhibitor therapy is challenged by the lack of informatic structures that enable the collection, analysis, and sharing of quantitative preclinical and clinical PET imaging data. To improve the repeatability of mouse tibia volume segmentation, Kushwaha et al. developed U-net-based deep learning models to streamline the bone segmentation for magnetization transfer MRI in a murine myelofibrosis model [37]. Spectral micro-CT with nanoparticle (NP) contrast enhancement can differentiate the lymphocyte (TL) burden in tumors. Allphin et al. are using a liposomal iodine (Lip-I) NP contrast agent for CT imaging in Rag2^−/−^ (TL deficient) and littermate control Rag2^+/−^ (TL present) mice, followed by radiomic analysis, to quantitatively assess TL burden [38].

Informatics remains a big challenge for co-clinical quantitative imaging. A major unsolved issue is how to manage and integrate the enormous amount of data that become available, across not only spatial and temporal scales, but also across species. Lewis et al. initiated a web-based analytical tool called Molecular and Imaging Response Analysis of Co-Clinical Trials (MIRRACL, https://miraccl.research.bcm.edu/, accessed on 28 April 2023) to simulate treatment response for co-clinical trials of TNBC treated with chemotherapy. MIRRACL seeks to cross-reference quantitative imaging features with omics data for the correlation of changes in vascularity and cellularity indices with changes in DNA, mRNA, and protein expression [39]. For the imaging in the preclinical setting, large volumes of image data are being generated that require critical data management tools. Pemmaraju et al. framed a web-based image registry and management tool for preclinical imaging research [40].

## 8. Future Perspective

The CIRP currently is in its sixth year, with two projects completed and eight projects to be completed during the 2023 to 2027 timeframe. These projects are complementary to each other in the context of relevant cancer issues, imaging methodologies, areas of expertise, and resource deliverables. All CIRP projects together provide a broad scope on how important cancer issues can be addressed using quantitative imaging methods in a co-clinical trial setting. The web resources developed through these projects can provide the broad cancer community with: (1) animal model (PDXs or GEMMs) methodologies to recapitulate biological features of human cancer, (2) imaging acquisition protocols to detect spatio-temporal tumor alterations in real time at molecular, cellular, functional, physiological, and anatomic levels, (3) imaging data processing tools to generate robust quantitative assessment for multi-cohort studies, and (4) research strategies to inform clinical trials by preclinical trials non-invasively and bridge the preclinical–clinical translational divide efficiently. The efforts of three WGs will focus on developing consensus across projects and establishing general guidance for the co-clinical quantitative imaging, paving an avenue toward the harmonization and standardization of existing and/or emerging quantitative imaging methods. The protocols and tools developed by the CIRP projects, and the guidance developed by the WGs can underpin core methods for future research. With some modifications and optimizations, these core methods can be extended to imaging diseases and therapies beyond the ones for which they were developed.

The CIRP is at an early stage of development and many unsolved issues emerge as the co-clinical imaging field matures. Examples are imaging drug kinetics, data heterogeneity effect, methodology interoperability, data format conversion, information integration from -omics to radiological images, sensitivity, and specificity improvement, and robust informatic tools. In the coming years, the CIRP network will continue to evolve and make further advances in these areas while delivering additional web resources to the cancer community. The CIRP continues to welcome academic investigators, who are interested in participating alongside CIRP investigators by joining the network as associate members, to contribute towards solving the many important remaining issues and to further advance the use of oncological imaging in the preclinical and clinical settings for co-clinical trials.

## Figures and Tables

**Table 1:** The CIRP projects and web-accessible resources.

Cancer Type	Disease	Animal	Therapy	Imaging	Web Resource	When *
Hematology	Myelofibrosis	GEMMs	Target therapy	MRI	UMICH: https://umu24cirp.med.umich.edu/	2024
Bone	Osteosarcoma	PDXs	Immunotherapy	MRI	Stanford: https://radweb.su.domains/cirp/	2026
Breast	TNBC	PDXs	Chemotherapy	PET/MRI	WUSTL: https://c2ir2.wustl.edu/	2022
	TNBC	PDXs	Chemotherapy	MRI	BCM/UTA/Stanford: https://miraccl.research.bcm.edu/	2024
	ER+/HER2-	PDXs	Hormone therapy	PET	WUSTL: https://c2ir2.wustl.edu/	2027
Colon	CRC	PDXs	Target/immunotherapy	PET	MDACC: https://www.mdanderson.org/research/departments-labs-institutes/programs-centers/predict.html	2023
Lung	NSCLC	GEMMs	Target therapy	PET	UW: https://sites.uw.edu/cocirp/	2026
Muscle	Sarcomas	GEMMs	RT/immunotherapy	CT, MRI	Duke: https://sites.duke.edu/pcqiba/	2022
Pancreas	PDAC	GEMMs	Target therapy	MRI	UPENN: https://pennpancreaticcancerimagingresource.github.io/	2023
Prostate	SCNC	PDXs	Chemotherapy	MRI	UCSF: https://coclinicalimaging.ucsf.edu/	2025

TNBC: Triple negative breast cancer, CRC: colorectal cancer, NSCLC: non-small cell lung cancer, ER: estrogen receptor, HER2: human epidermal growth factor receptor 2, PDAC: pancreatic ductal adenocarcinoma, and SCNC: small cell neuroendocrine prostate cancer. * Project completion time. All URLs in this table are accessed on 20 April 2023.

## Abbreviations

2D	two-dimensional
3D	three-dimensional
ADC	apparent diffusion constant
AMCT	animal models and co-clinical trials
BM	bone marrow
CCDB	co-clinical database
CIMI	co-clinical imaging minimal information
CIRP	co-clinical imaging research resource program
CRC	colorectal cancer
CT	computed tomography
CTEP	cancer therapy evaluation program
CXCR2	C-X-C motif chemokine receptor 2
DCE	dynamic contrast enhanced
DICOM	digital imaging and communications in medicine
DNA	deoxyribonucleic acid
EGFR	epidermal growth factor.
EPI	echo planar imaging
ER	estrogen Receptor
^18^F-FDG	^18^F-fluorodeoxyglucose
FF	fat fraction
^18^F-FFNP	^18^F-fluoro-furanyl-norprogesterone
^18^F-FSPG	(4*S*)-4-(3-^18^F-fluoropropyl)-L-glutamic acid
GEMMs	genetically engineered mouse models
^18^F-Gln	^18^F-4-fluoro-glutamine
HER2	human epidermal growth factor receptor 2
HP	hyperpolarized
IADP	imaging acquisition and data process
IDC	imaging data common
IMOR	informatics and outreach
ITCR	informatics technology for cancer research
Lip-I	liposomal iodine
MIRRACL	molecular and imaging response analysis of co-clinical trials
MMHCC	mouse models of human cancers consortium
MTR	magnetization transfer ratio
MRI	magnetic resonance imaging
MRSI	magnetic resonance spectroscopy imaging
NIFTI	neuroimaging informatics technology initiative
NP	nanoparticle
NSCLC	non-small cell lung cancer
OMF	oncology model forum
PDAC	pancreatic ductal adenocarcinoma
PD-L1	anti-programmed death-ligand 1
PDMC	patient derived models of cancer
PDMR	patient-derived model repository
PDX	patient derived xenograft mouse model
PDXnet	PDX development and trial centers research network
PERCIST	PET response criteria in solid tumors
PET	positron emission tomography
PIPs	preclinical Imaging Protocols
PIVOT	pediatric in vivo testing program
PPTC	pediatric preclinical testing consortium
QA/QC	quality assurance/quality control
QIN	quantitative imaging network
RCC	renal cell carcinoma
RNA	ribonucleic acid
SCNC	small cell neuroendocrine prostate cancer
SNR	signal to noise ratio
SOP	standard operating procedure
SPORE	specialized program of research excellence
TAM	tumor-associated macrophage
TCIA	the cancer imaging archiving
TL	tumor lymphocyte
TNBC	triple negative breast cancer
WG	working group
